# Modulation of Plant Defense System in Response to Microbial Interactions

**DOI:** 10.3389/fmicb.2020.01298

**Published:** 2020-07-03

**Authors:** Resna Nishad, Talaat Ahmed, Vattakandy Jasin Rahman, Abdul Kareem

**Affiliations:** ^1^Department of Biological and Environmental Sciences, College of Arts and Science, Qatar University, Doha, Qatar; ^2^Environmental Science Centre, Qatar University, Doha, Qatar; ^3^T.K.M. College of Arts and Science, Kollam, India; ^4^School of Life and Environmental Sciences, The University of Sydney, Sydney, NSW, Australia

**Keywords:** plant immunity, innate immunity, microbe-associated molecular pattern-triggered immunity, effector-triggered immunity, mycorrhiza-induced resistance, beneficial microbes

## Abstract

At different stages throughout their life cycle, plants often encounter several pathogenic microbes that challenge plant growth and development. The sophisticated innate plant immune system prevents the growth of harmful microbes via two interconnected defense strategies based on pathogen perception. These strategies involve microbe-associated molecular pattern-triggered immunity and microbial effector-triggered immunity. Both these immune responses induce several defense mechanisms for restricting pathogen attack to protect against pathogens and terminate their growth. Plants often develop immune memory after an exposure to pathogens, leading to systemic acquired resistance. Unlike that with harmful microbes, plants make friendly interactions with beneficial microbes for boosting their plant immune system. A spike in recent publications has further improved our understanding of the immune responses in plants as triggered by interactions with microbes. The present study reviews our current understanding of how plant–microbe interactions can activate the sophisticated plant immune system at the molecular level. We further discuss how plant-microbe interaction boost the immune system of plants by demonstrating the examples of *Mycorrhizal* and *Rhizobial* association and how these plant-microbe interactions can be exploited to engineer disease resistance and crop improvement.

## Introduction

Plants encounter a wide range of microorganisms throughout their lifetime, and their interactions with these microorganisms can be either beneficial or deleterious, resulting in the establishment of mutualistic or pathogenic interactions, respectively (Thrall et al., 2007; Rodriguez et al., 2019). To respond to various beneficial and pathogenic microorganisms, plants can modulate their innate immune system based on the mechanism induced by the microbes and exhibit appropriate responses (Pieterse et al., 2014). Plants possess a sophisticated immune response strategy that can be expressed either constitutively or following a microbial challenge. Once a microorganism overcomes these protectant barriers, it establishes a consistent interaction with the plant, leading to either a beneficial association or a disease.

Mutualistic associations can induce immune responses against other microorganisms. In addition, detrimental microbial associations trigger immune system induction but against themselves. Although various pathogens exist in the surrounding soil, water, and air, the total loss of a crop to disease is not common. This reflects the plant’s defense systems and natural biocontrol processes to control pathogens. Recently, the role of beneficial non-pathogenic microbes in the defense priming of host plants has been reported (Dey et al., 2014; Martínez-Hidalgo et al., 2015; Singh et al., 2016), which suggest that plant immunity is induced if a successful interaction is achieved via non-pathogenic microbes. Mycorrhizal and rhizobial associations are important examples of immune responses induced by beneficial microbial associations. However, detailed studies are warranted to understand how the mutualistic plant–microbe association induces plant immunity to develop new disease control measures in the field of agriculture. The use of beneficial microbes to induce plant defense response against pathogenic microbes will be an ecofriendly alternative to hazardous chemical pesticides in disease management. This review discusses various types of plant defense responses modulated by plant interactions with diverse microbial communities and outlines the possible strategies to boost plant immunity.

## Plant Defense Responses

### Innate Immunity

**FIGURE 1:**
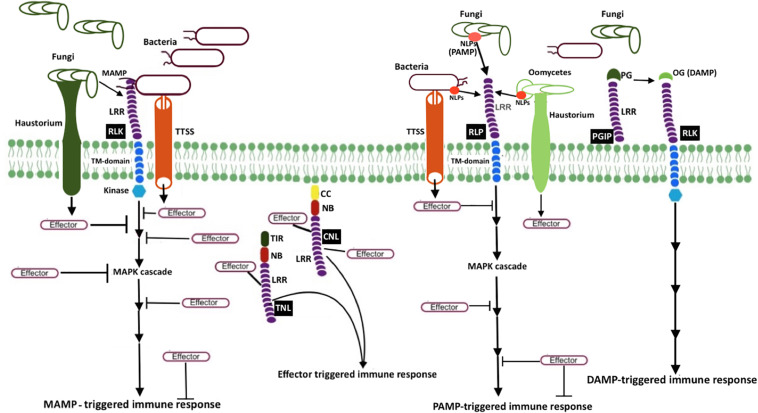
Schematic representation of microbial resistance in plants MAMP (e.g., bacterial flagellin flg22 and fungal chitin) recognition by receptor-like kinases (RLK; a PRR) triggers mitogen-activated protein kinase (MAPK) cascades, eventually resulting in MAMP-triggered immunity (MTI). Another PRR, receptor-like protein (RLP, e.g., RLP23), perceives MAMP or pattern-associated molecular patterns (PAMP), and induces pattern-triggered immunity (PTI). Necrosis and ethylene–inducing peptide 1 (NEP1)–like proteins (NLPs) are examples of PAMP, which are recognized by RLP23. To counteract plant defense responses, pathogen release effectors into plant cells. Bacteria secrete and deliver effector proteins into plant cells via type III secretion system (T3SS), whereas fungi and oomycetes secrete effectors via haustoria. Once plant resistance protein coiled–coil (CC) NLR (known as CNL) and Toll-interleukin-1 receptor (TIR) NLR (known as TNL) recognize the effector activity inside the cell, effector-triggered immune responses (ETI) will be induced. The extracellular LRR protein—polygalacturonase-inhibiting protein (PGIP)—interacts with microbial polygalacturonase (PG), which slow down the plant pectin degradation and results in the formation of oligogalacturonides (OG), a damage-associated molecular pattern (DAMP). DAMP perception by PRR triggers DAMP-triggered immune responses (DPI).

Plants block a majority of microbes at the front line by a non-host resistance strategy. It involves physical barriers such as waxy cuticles, rigid cell walls, and antimicrobial secondary metabolites. The pathogens that successfully overcome these barriers have to encounter the efficient plant immune system terminating the progression of microbial colonization. Unlike mammals, plants do not have a somatic adaptive immune system with mobile defender cells. Instead, they depend on each cell exerting innate immunity, with systemic signals emerging from the infected cells and the ability of plant cells to remember previous infections (Reimer-Michalski and Conrath, 2016). The currently adopted zig-zag coevolutionary model describes two branches of molecular defense strategies (Jones and Dangl, 2006). The first branch utilizes the recognition of the microbe or pathogen or damage-associated molecular patterns (MAMP/PAMP/DAMP) through plant cell surface-anchored pattern recognition receptors (PRRs) to induce a set of responses such as MAMP-triggered immunity (MTI), PAMP-triggered immunity (PTI), and DAMP-triggered immunity collectively referred to as pattern−triggered immunity (PTI; Saijo et al., 2018). The second branch utilizes the recognition of microbial effectors, the virulence factors that suppress MTI, through resistance (R) proteins to initiate effector-triggered immunity (ETI). The activation of these immune responses triggers a cascade of complex signaling events, leading to suppression of pathogen attacks (Figure 1).

#### MTI

To understand the first branch of immune response, we will focus on MTI. The plant membrane receptors—PRRs—recognize MAMPs to induce MTI, thereby resulting in rapid calcium influx, reactive oxygen species (ROS, also known as the oxidative burst) accumulation, mitogen-activated protein kinase (MAPK) phosphorylation cascades, cell wall alterations, and defense gene expressions (Windram and Denby, 2015; de Lorenzo et al., 2018). Bacterial flagellin, elongation factor, fungal chitin, and lipopolysaccharides (LPS) are examples of conserved MAMPs present in microbes.

Given that MTI is an important immune response in plants, great attention has been paid to understand the recognition mechanism of MAMPs by PRRs and the complex network underlying signaling events. PRRs usually are plasma membrane-bound proteins such as receptor-like kinases (RLKs) or receptor-like proteins (RLP) with extracellular domains. FLAGELLIN-INSENSITIVE 2 (FLS2) and elongation factor-Tu (EFR) are PRRs that recognize flagellin epitope (flg22) and bacterial elongation factor-Tu epitope (elf18), respectively (Bauer et al., 2001; Zipfel et al., 2006). Chitin elicitor receptor kinase 1 (CERK1) is another example of a PRRs that perceives fungal chitin and bacterial peptidoglycan (Miya et al., 2007; Willmann et al., 2011).

FLAGELLIN-INSENSITIVE 2, belonging to the RLK family, is a well-studied PRR in *Arabidopsis*. FLS2 requires G protein for innate immune signaling (Liang et al., 2016). Trusov and Botella (2012) have reported the involvement of G protein in plant defense, and since then researchers have vested interest toward G protein. Unlike animal G proteins, plant G proteins are self-activating and thus independent of G protein-coupled receptors, they are coupled to receptor kinases, including FLS2 (Urano and Jones, 2014; Liang et al., 2016). Upon perceiving flg22, FLS2 forms a dynamic complex by recruiting the coreceptor—BRI1-associated receptor kinase (BAK1), receptor-like cytoplasmic kinase Botrytis-induced kinase 1(BIK1), and G protein—that activates defense responses (Liang et al., 2016). Liang et al. (2018) recently reported that the regulator of G protein signaling 1(RGS1), a GTPase accelerating protein, maintains an inactive state of G proteins in complex with FLS2. Flg22-induced activation of FLS2 leads to BIK1-mediated phosphorylation of RGS1, which in turn results in the dissociation of RGS1 from the FLS2–G protein complex. Upon relieving the RGS1-mediated repression of G protein, positive regulation of immune signaling occurs.

Upon MAMP perception, ROS burst occurs rapidly via RBOHD, an NADPH oxidase. BIK1 directly phosphorylates RBOHD to prime flg22- induced ROS (Li L. et al., 2014). RBOHD produces membrane-impermeable superoxide (O${}_{2}^{-}$), and superoxide dismutase (SOD) converts this O${}_{2}^{-}$ into H_2_O_2_ in apoplasts (Podgórska et al., 2017). H_2_O_2_ enters into the cytosol and induces cytosolic Ca^2+^ elevation (Yuan et al., 2017).

Influx of extracellular Ca^2+^ in the cytosol (also known as Ca^2+^ burst that is positively regulated by BIK1) is an earliest response to MAMP perception (Jeworutzki et al., 2010). Ca^2+^ activates phospholipase C that releases a downstream secondary messenger that leads to further Ca^2+^ release (Li L. et al., 2014). Ca^2+^ binds to a sensor molecule (calmodulin or Ca^2+^-dependent protein kinases). The activated sensor molecule phosphorylates the protein kinase, leading to either the expression of the defense gene or activation of the enzyme for metabolite production. Ca^2+^ influx activates the H^+^/K^+^ ion fluxes and also Cl^–^, K^+^, and NO${}_{3}^{-}$ efflux which leads to extracellular alkanization and depolarization of the plasma membrane (Jeworutzki et al., 2010). Even though Ca^2+^ induced immune signaling response has been studied, the underlying mechanisms of PRR induced Ca^2+^ entry remained unclear until the recent study by Tian et al. (2019). In plant cell, calmodulin blocks CNGC2-CNGC4 Ca^2+^ channels and remains closed in the resting state. Upon pathogen attack, BIK1 phosphorylates C-terminal cytosolic domain of CNGC4 and activates the channel, which results in the increased cytosolic calcium concentration (Tian et al., 2019).

**FIGURE 2:**
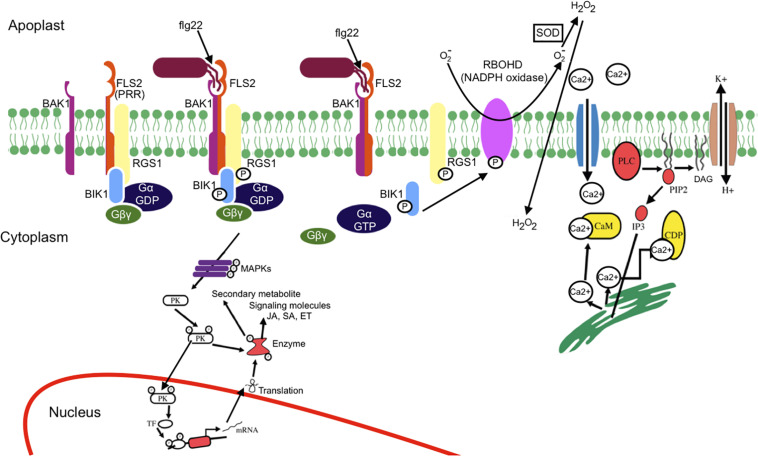
Schematic representation of MAMP-PRR-induced signaling events. Upon flg22 (MAMP) perception by FLS2 (PRR), an immune response complex is rapidly formed. The receptor complex (FLS2, BIK1, BAK1, and G protein) perceives flg22 and induces active FLS2–BAK1 receptor complex formation and BIK1-dependent phosphorylation of RGS1, which results in dissociation of RGS1 from G proteins and FLS2. The release of RGS1 from G protein leads to the spontaneous conversion of Gα^GDP^ into Gα^GTP^. Ca^2+^ influx occurs as an earliest response of MAMP perception. Ca^2+^ influx activates H^+^/K^+^ exchange. Ca^2+^ activates phospholipase C that releases a downstream secondary messenger, leading to further release of Ca^2+^. The activation of phospholipase C (PLC) either by G protein or Ca^2+^ leads to the breakdown of phosphatidyl-4, 5-bisphosphate into diglyceride (DAG) and inositol 3-phosphate, eventually causing structural instability. BIK1 phosphorylates the NADPH oxidase (RBOHD), leading to reactive oxygen species burst. The activated sensor molecule phosphorylates the protein kinase that leads either to expression of the defense gene or activation of the enzyme for metabolite production. MAPK cascade leads to phosphorylation of transcription factor (TF) and activation of salicylic acid (SA), jasmonic acid (JA), and ethylene (ET) signaling. Mitogen-activated protein kinase (MAPK) cascade also result in production and secretion of antimicrobial compounds.

Mitogen-activated protein kinase activation occurs in a few minutes (2–3 min after flg22 perception) within the same time frame as the Ca^2+^ burst. MAPK activation leads to phosphorylation of ethylene (ET)-dependent transcription factor (TF), activation of ET responsive genes, and synthesis of ET (Jagodzik et al., 2018). Bigeard et al. (2015) have extensively reviewed MAPK-triggered immunity in plants. MAPK activation leads to phosphorylation of several TFs that regulates several genes such as those involved in salicylic acid (SA), jasmonic acid (JA), and ET signaling as well as antimicrobial compound production. Finally, this complex signaling network results in plant-induced defenses. FLS2 receptor complex-induced signaling mechanism is illustrated in Figure 2. For clarity, *Arabidopsis thaliana* FLS2 has been considered as a model.

#### ETI

To counteract MTI, pathogen releases effector protein into the host cell that leads to effector-triggered susceptibility. Intracellular immune receptors, namely nucleotide binding (NB) leucine rich -rich repeat (LRR) receptor (NLR) proteins, encoded by resistant genes recognize these effectors or their activity and induce ETI (Dangl and Jones, 2001). NLR proteins belong to signal transduction adenosine triphosphatases (ATPases) with numerous domains (STANDs) including a conserved tripartite domain structure that contains a conserved central NB and oligomerization domain, a C-terminal LRR domain, and a non-conserved N-terminal domain. The central NB and oligomerization domain can be further divided into a helical domain (HD1), a winged-helix domain (WHD), and an NB domain (NBD). NLR proteins are divided into two groups based on their N-terminal domain: coiled coil (CC)–NLRs and Toll-interleukin-1 receptor (TIR)–NLRs. CC-containing NLR are called as CNLs and TIR domain containing NLRs known as TNLs.

The activation of plant NLRs cease pathogen proliferation by inducing different immune responses including Ca^2+^ signaling, nitric oxide and ROS production, alteration in membrane trafficking, transcriptional reprogram of defense genes, and program cell death called hypersensitive response (Grant et al., 2000; Gao et al., 2013; Caplan et al., 2015). JA and SA accumulation, antimicrobial molecule and hydrolytic enzyme production, and callose deposition at the site of infection are also detected as a result of effector perception.

Plant NLRs function as molecular switch with ADP-bound autoinhibited “off” state and ATP-bound activated “on” state (Hu et al., 2013). Intracellular interaction between CC or TIR and LRR keep the NLR protein in an inactive state. Direct or indirect recognition of effector triggers opening of NLR protein, alters intramolecular interaction, relieves inhibition, and causes exchange of ADP for ATP, followed by activation of NLR protein which triggers downstream signaling (Zhang et al., 2017; Kourelis and van der Hoorn, 2018). NLR mutation and swap experiments support the key role of variable C-terminal LRR domain in the effector recognition (Krasileva et al., 2010; Ravensdale et al., 2012).

NLR induce ETI either by directly recognizing effectors or indirectly recognizing host proteins that have been modified by effector activity (Dodds et al., 2006; Ade et al., 2007; Wang et al., 2019a, b). In indirect recognition model, NLR protein either recognize effector modified host target protein known as guardee, that is bound to and monitored by NLR protein, or recognize effectors modified plant decoy protein that mimic host target protein (Chung et al., 2014; Li M. et al., 2014; Ntoukakis et al., 2014). Many identified guardee have key immune related function such as signaling whereas decoy protein has no measurable resistant function.

**FIGURE 3:**
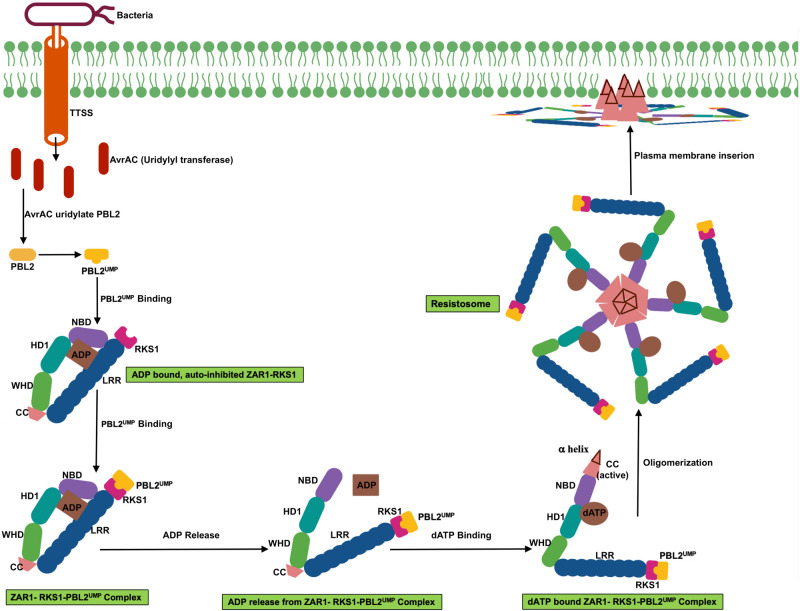
Schematic representation of transition of ZAR1 (a CC-NLR) from inactive to active oligomeric state. ZAR1 maintain inactive state in cells through the contact of pseudokinase RKS1 and ADP molecule. AvrAC effector from the bacteria uridylate PBL2 (a decoy protein) to PBL2^UMP^. Uridylation enable binding of PBL2^UMP^ to RKS1, which cause conformational change, NBD rotate outward, and consequently ADP release from ZAR1. Then ATP or dATP bind to ZAR1- RKS1-PBL2^UMP^ complex, which further results in second conformational change, fold switching of CC domain. Oligomerization of this complex form pentameric ZAR1- RKS1-PBL2^UMP^. As a consequent of structural change, amphipathic α helix release from very N-terminal of ZAR1 and form funnel shaped structure. This structure may promote ZAR1 association with plasma membrane and result in cell death by affecting plasma membrane integrity.

Even though plant NLRs induce program cell death similar to animal NLR, the signaling mediator such as caspases have not been identified in plants. However, it was not known whether plant NLR oligomerize upon activation. The recent report of *Arabidopsis* coiled-coil (CC)–NLR protein HOPZ-ACTIVATED RESISTANCE 1 (ZAR1) forming oligomeric state or the “resistosome” provided the insight of plant NLR function (Wang et al., 2019a, b). ZAR1 indirectly recognizes the bacterial effector proteins through an association with pseudokinase known as resistance-related kinase 1 (RKS1). ZAR1-RKS1 complex indirectly recognizes *Xanthomonas campestris* pv. *campestris* effector AvrAC (uridylyl transferase). AvrAC first uridylylates PBS1-like protein 2 (PBL2), a receptor-like cytoplasmic kinases (RLCK), it is a decoy protein. Uridylylated PBL2 (referred as PBL2^UMP^) binds to the ZAR1-RKS1 complex which triggers conformational change in NB domain and enables ADP to release from inactive ZAR1 which results in nucleotide depleted ZAR1 (ZAR1- RKS1-PBL2^UMP^ complex). The ZAR1- RKS1-PBL2^UMP^ complex is still in inactive state; hence, a second signal is required for its activation. ATP or dATP activates ZAR1- RKS1-PBL2^UMP^ complex and acts as a best candidate molecule to trigger the second signaling step that leads to the oligomerization of the NLR protein. ATP or dATP binding induces second conformational change which results in NBD rearrangement and restructuring of CC domain. Then the complex forms a wheel-like pentameric active “resistosome.” A funnel-shaped structure formed from the α helices of oligomeric CC domains is essential for plasma membrane association activities and immune signaling. Plasma membrane associated resistosome can affect integrity of plasma membrane or ionic homeostasis (Wang et al., 2019a, b; Figure 3).

## Systemic Acquired Resistance (SAR)

Previously challenged (primed) plants exhibit a greater resistance against subsequent challenges via a phenomenon known as systemic acquired resistance (SAR). Encountering with the pathogen for the second time in the SAR state activates an effective immune response (Conrath, 2006) and possibly provides long-lasting protection ranging from weeks to a month and sometimes throughout an entire season. Tissue necrosis caused by the plant pathogen induces SAR either as a part of HR or as a symptom of a disease. One of the characteristic features of SAR is the spread of increased resistance to the distal uninoculated plant organs on the challenged plant (Adam et al., 2018). Another feature of SAR is its activity against a wide and distinctive range of pathogens, including bacteria, fungi, oomycetes, and viruses. SAR is evidence for the existence of plant memory.

Metabolites such as SA and pipecolic acid (Pip) are vital for SAR, with each one activating different sets of defense-related genes (Bernsdorff et al., 2016). Recent research demonstrates that pathogen induced L-lys catabolic pathway, which generates the N-hydroxypipecolic acid (NHP) from Pip, is central of plant SAR, and flavin-dependent-monooxygenase1 (FMO1) is a key regulator in the pathway (Chen et al., 2018; Hartmann et al., 2018). NHP is found systemically accumulated upon microbial attack. Exogenous application of NHP in *Arabidopsis* is also found inducing resistance against bacteria and oomycetes. Hence metabolic engineering of pipecolic acid pathway in plants can be a promising strategy for enhanced disease resistance (Chen et al., 2018).

Several metabolites that are involved in long-distance signaling have been identified. Methyl esters of SA (MeSA), dicarboxylic acid, azelaic acid (AzA), and abietane diterpenoid dehydroabietinal (DA) are some examples of such types of metabolites. It is known that SA is needed to establish systemic resistance in distal tissues, and MeSA and DA promote SA accumulation in distal leaves. AzA and Pip primes the faster and stronger accumulation of SA (Shah and Zeier, 2013). The first convincing evidence for the indispensable role of SA in SAR came from studies on transgenic tobacco and *Arabidopsis* plants that constitutively express bacterial SA hydroxylases. These plants were unable to accumulate elevated concentrations of SA and consequently could not secure systemic resistance following challenge with necrotizing pathogens (Gaffney et al., 1993; Delaney et al., 1994) possibly because of the destruction of the SA signal. Pathogen-induced SA signal travels across plant cells and activates signal transduction, eventually activating SAR-inducing gene expression and increasing resistance to subsequent infection (Gruner et al., 2013).

## Host Immunity Failure Leading to Plant Diseases

Because each plant species has the natural ability to recognize and react to pathogen attacks and to resist most potential pathogens (León and Montesano, 2013), plant diseases are not common in natural ecosystems, particularly in the agroecosystem. Then, what causes host immunity failure to precipitate disease? Once a pathogen overcomes the host plant’s defenses, it can harness the plant’s primary production and reproduce, and in most cases, this leads to striking disease symptoms (Jones and Takemoto, 2004). Conversely, when the plant resistance framework restricts the growth of pathogens, no disease symptoms occur. Immune system failure permits further entrance into the host plants by the attacking pathogens. Pathogens have evolved to develop effector proteins and other small molecules to stifle the MAMP-activated defenses (Jones and Dangl, 2006; Zheng et al., 2012) so that the pathogen establishes effector-triggered susceptibility by suppressing MTI (Pel and Pieterse, 2013). Effectors can also manipulate host target proteins to disable the plant immune system. Such examples include an effector from phytoplasma bacteria that binds to host Teosinte branched 1/Cincinnata/proliferating cell factor (TCP)/TFs to inhibit JA synthesis (Sugio et al., 2011). Effectors secreted from oomycetes inhibit host extracellular defense enzymes and suppress host defense by interfering with plant processes inside the host cell, with the AVR3a effector from *Phytophthora infestans* stabilizing ubiquitin ligase to prevent hypersensitive-like host cell death, allowing further cell ingress by the pathogen (Stassen and Van den Ackerveken, 2011). Transcriptional reprogram of host plants by pathogen effector molecules to suppress immunity has also been reported, with transcription activator-like (TAL) effectors from *Xanthomonas oryzae* pv. *oryzae* binding specifically to promoter sequences called TAL effector binding elements in rice *Xa5* genes to activate transcription of susceptibility genes such as *OsSWEET11* and *OsSWEET14* (Mak et al., 2013).

In addition to host immunity failure, processes such as attraction to and attachment of the pathogen to the host plant, allowance of room for infection structures in the plant cell, and provision of food for the pathogen can contribute to increased plant susceptibility to pathogen (Lapin and Van den Aclerverken, 2013). Plant signals promote pathogen development, and this has been widely studied in different plants. Gene expression studies on germinating spores of *Colletotrichum higginsianum*, a hemibiotrophic fungus, revealed the effects of host plant signals on early pathogen development. Transcription of more than 1,700 genes was induced upon *C. higginsianum* spore attachment onto the host plant surface compared with that upon attachment onto an artificial polystyrene surface (O’Connell et al., 2012). Some plant-derived chemicals such as alcohols, aldehydes, fatty acids, and flavonoids attract plant pathogens, promote their attachment, and finally induce the formation of penetration structures known as appressoria (Lapin and Van den Aclerverken, 2013). The release of flavonoids from the plant roots attracts soil-borne pathogens such as *Phytophthora* spp. (Morris and Ward, 1992). Plant hormones such as ET from ripening fruits can induce spore germination and appressorium formation in *C. gloeosporioides* (Kolattukudy et al., 1995). Thus, the plant signal-motivated attraction of pathogenic microbes to the plant provides a favorable environment for pathogens to cause diseases.

Some pathogens manipulate the host cellular machinery by producing toxic substances such as HC toxin that is a histone deacetylase inhibitor produced by the phytopathogenic fungus *Cochliobolus carbonum*, which reprograms the host transcriptional response to pathogen challenge in maize, causing ineffective immune defense response (Walley et al., 2018). Before practicing the transfer of beneficial microorganisms to the field, extensive studies must be conducted to ensure that beneficial microorganisms do not acquire such a toxin gene from a pathogen by horizontal gene transfer.

## Mutualistic Plant–Microbial Association and Induced Plant Immune Response

In addition to innate immunity and SAR, plants develop another type of induced immune response via mutualistic association with friendly microbes. This response is achieved by providing a shelter for friendly beneficial microbes within the plant body, thus gaining resistance against harmful microbes. The beneficial microbes synthesize toxins against invading harmful microbes and protect the plant from pathogenic diseases. This type of plant–microbe interaction is often called a symbiotic association. Associations with mycorrhiza, rhizobium, and endophytic microbes are some examples of mutualistic plant–microbe interactions and induce plant immune response.

### Mycorrhizal Association and Its Role in Plant Immune Response

Plant–mycorrhizal association is one of the examples of mutualistic association that plays an important role in stimulating plants immune response against soil-borne diseases and pests (Sanchez-Bel et al., 2016). In response to colonization by arbuscular mycorrhizal fungi (AMF), plants develop an enhanced defensive strategy known as mycorrhiza-induced resistance (MIR; Cameron et al., 2013). AMF suppresses plant diseases and pests through induced systemic resistance (ISR; Jung et al., 2012; Song et al., 2015). MIR shares features of pathogen-induced SAR and non-pathogenic rhizobacterium- ISR.

From the plant’s perspective, the mycorrhizal fungus is an alien “non-self” organism, and plants may recognize it as a pathogen and exhibit an immune response, which the fungus then has to overcome to establish a successful interaction. Given that AMF lives within the plant, the colonization process is similar to infection by a biotrophic pathogen and hence the initial defense responses induced against these two classes of microorganisms are similar (Pieterse et al., 2012). Increases in SA production occur in the root following AMF colonization, along with accumulation of defensive products such as hydrolytic enzymes and ROS as well as activation of the phenylpropanoid pathway, albeit at a lower level than that during the plant–pathogen interaction (Fester and Hause, 2005; Scheler et al., 2013).

Compared with the plant–pathogen interaction, the mutualistic response generated during AMF colonization is spatiotemporally limited, suggesting that control operates over the immune response and the establishment of mutualism (Garcia-Garrido and Ocampo, 2002). Nevertheless, increases in SA concentration have been reported to have a negative effect on AMF colonization (de Roman et al., 2011). Therefore, for successful establishment of an AMF association, inhibition of some SA-regulated responses is essential, as described for other mutualistic associations (Dumas-Gaudot et al., 2000). In this manner, the secretory effector proteins from AMF interfere with the host plant immune system by suppressing SA-dependent defense reactions (Kloppholz et al., 2011). The upregulation of other phytohormones, such as JAs, also plays a central role in the establishment and maintenance of the AMF mutualistic association (Hause et al., 2007). Once a successful association is established between the AMF and the plant, the induced JA plays an important role in MIR (Yogendra et al., 2015). AMF-colonized plants are more resistant to necrotrophic pathogens (Pozo et al., 2009) and more susceptible to biotrophic pathogens, and these observations can be explained by the role of JA in defense against necrotrophs and of SA in defense against biotrophs (Robert-Seilaniantz et al., 2011). In AMF associations, SA-dependent responses are suppressed, whereas JA-dependent responses are activated.

After establishing a successful association with a plant, AMF recruit root-associated bacteria such as plant growth-promoting rhizobacteria to upregulate immune responses against pathogens. Cameron et al. (2013) proposed a four-phase spatiotemporal model to describe the overall mechanism involved in the mycorrhizal association and MIR. The first phase is the release of plant root exudates such as sugars, amino acids, phenolic compounds, and other secondary metabolites, which attract soil microorganisms to the roots. More importantly, root exudates comprising strigolactones have the ability to recruit AMF to plant roots (Akiyama et al., 2005). In the second phase, the plant exerts an immune response against AMF. This immunity induction is based on the recognition of the AMF’s MAMPs using the plant’s PRRs, thereby enhancing SA accumulation and triggering the immune response (Zhang and Zhou, 2010).

The next phase is the suppression of plant immunity by mycorrhizae and the recruitment of mycorrhizosphere bacteria. Immunity suppression by AMF is similar to that in the process of plant–pathogenic fungus association, wherein pathogenic fungi suppress plant immunity by secreting specific effectors and subsequently establishing a successful infection (de Jonge et al., 2011). Furthermore, AMF exerts immune suppression through calcium/calmodulin kinase DMI3, which represses early-acting defense genes (Siciliano et al., 2007). AMF reportedly induces the production of the plant hormone abscisic acid (ABA) to boost its own colonization of the host root system via suppressing the SA-dependent defense response (Ton et al., 2009). Although SA induction is suppressed during successful AMF colonization, the fact that the initial SA response can lead to production of long-distance SAR signals could be the basis of SA-based defense (Heil and Ton, 2008; Conrath, 2011).

**FIGURE 4:**
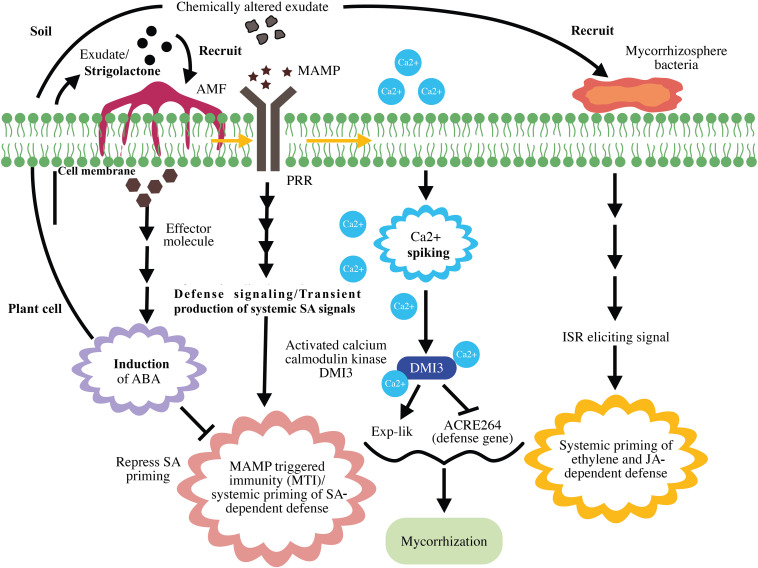
Schematic representation of mycorrhization and mycorrhiza-induced resistance. Strigolactone in plant root exudates recruits arbuscular mycorrhizal fungi (AMF) to the plant. Upon recognition of mycorrhizal microbe-associated molecular patterns (MAMPs), pattern recognition receptors (PRR) induce MAMP-triggered immunity (MTI) and systemic priming of salicylic acid (SA)-dependent defenses. Mycorrhizal effector-induced ABA represses SA priming and suppresses MTI. MAMP-triggered calcium spiking results in the activation of Ca^2+^/calmodulin kinase DMI3, which represses early defense gene expression and promotes mycorrhization. The successful mycorrhizal association results in chemical alteration of exudates, rhizobacterial recruitment, and mycorrhizosphere development. Mycorrhiza-induced systemic resistance is the result of ethylene (ET)- and jasmonic acid (JA)-dependent defenses exerted by mycorrhizosphere bacteria.

The last phase of the four-phase spatiotemporal mycorrhizal model is mycorrhizosphere development and ISR. The successful mycorrhizal association enhances the transport of photosynthates to the roots and influences the sugar-dependent signaling pathways (Smith and Smith, 2011). Taken together, this activity and improved phosphate uptake by mycorrhizae change the composition of root exudates. These changes enhance rhizobacterial recruitment and subsequently mycorrhizosphere development (Jung et al., 2012). Similar to that with AMF, rhizobacteria comprise MAMPs that activates MAMP-induced immune responses (Berendsen et al., 2012). Thus, MIR is the result of a direct plant response against arbuscular mycorrhizal colonization and an indirect response against rhizobacteria, which inhabit the mycorrhizosphere (Cameron et al., 2013), indicating the involvement of ISR induced by bacteria in MIR. Systemic priming of both ET- and JA-inducible defenses has frequently been reported in both ISR and MIR (Van der Ent et al., 2009). Cameron et al. (2013) proposed that JA-dependent defense priming during MIR is the result of ISR elicited by rhizobacteria. A schematic representation of mycorrhization and MIR is presented in Figure 4.

### Rhizobial Associations and the Role of Rhizobacteria in Plant Immune Response

Plants recognize beneficial rhizobial microbes, including symbiotic and non-symbiotic microbes, as alien just as they may recognize mycorrhizae as pathogens. Unless these microbes actively interfere with the plant immune system, they cannot establish successful associations with the host plant. Bacterial flagellin (MAMP) induces a defense response during the *Rhizobium*-legume mutualism that does not persist for more than 24 h due to a strong suppressive mechanism exerted by the bacteria. In mutualistic rhizobial associations, rhizobia induce MTI, which is found to be suppressed during the later stages of root nodule formation and the underlying mechanism for which has not yet been clearly defined. The suppression of plant immunity during the initial phase of mutualism has been recently reported (Zamioudis and Pieterse, 2012). Succinoglycan, an exopolysaccharide (EPS) produced by bacteria, is proposed to suppress plant immune defense during the initial phase of mutualism (Jones et al., 2008). EPS suppresses MTI by chelating calcium (Aslam et al., 2008), an important signal molecule in MTI.

**FIGURE 5:**
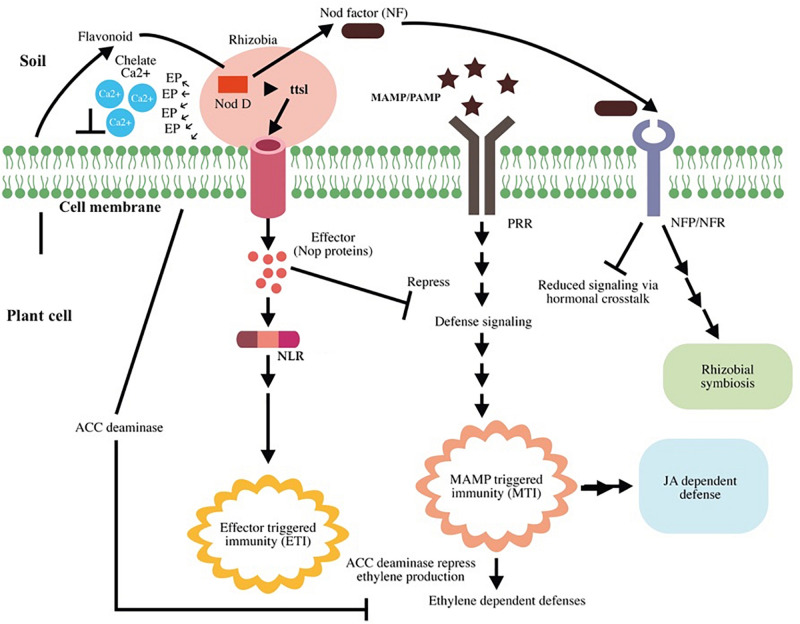
Schematic representation of rhizobial mutualism and plant immunity modulation. Root exudates recruit *Rhizobium* to the plant root. Flavonoids activate the nod gene transcription factor NodD and enhance nod factor (NF) production and *ttsI* expression. *TtsI* expression results in effector molecule production that suppresses the host defense signals. Some effector molecules cause effector-triggered immunity (ETI) in the host. Intracellular receptor for NLR recognizes the effector and activates ETI. NF induces root nodule formation and successful *Rhizobium*–plant association after interacting with their host receptors (Nod factor receptors, NFRs or Nod factor perception, NFPs). Upon recognition of rhizobial flagellin [microbe-associated molecular patterns (MAMPs)], pattern recognition receptors (PRR) induce microbe-associated molecular pattern-triggered immunity (MTI); however, NF partially suppresses microbe-associated molecular pattern-triggered immunity (MTI) by affecting salicylic acid (SA) production via hormonal cross talk. The plant recognizes *Rhizobium* as a pathogen and induces MTI, which results in ethylene (ET)-, and jasmonic acid (JA)-dependent defenses. Bacteria secrete 1-aminocyclopropane-1-carboxylic acid (ACC) deaminase enzyme to target the precursor of ET and suppress ET production, thereby affecting ethylene-induced defense. *Rhizobium* association does not affect the JA-dependent signaling pathway, indicating its major role in induced systemic resistance (ISR) and conferring immunity in distal as well as local cells.

Root nodule is the result of the interaction between the flavonoid excreted from the host plant and NodD, a rhizobial TF that induces the rhizobial nod gene (nodule gene). This nod gene is required for lipochitooligosaccharide (Nod factor, NF) synthesis. NF induces root nodule formation; NFs and their host receptors (Nod factor receptors, NFRs or Nod factor perception, NFPs) are required for nodule organogenesis and root hair infection (Kouchi et al., 2010). Rhizobium bacterium infects the legume through root hair and infection threads; furthermore, NF helps root hair to re-enter the cell cycle and enhance the spread of rhizobia in the cortex. After infection, root nodule harbors a striking number of rhizobia in root nodules (Oldroyd et al., 2011). Beyond organogenesis, the nodulation (nod) factor (NF) plays a crucial role in immune suppression, thereby partly suppressing MTI (Liang et al., 2013). NF may affect the strength of local induction of the SA-signaling pathway via hormonal cross talk, given that the presence of NF increases the levels of cytokinins and auxins in cortical cells (Oldroyd and Downie, 2008; Pieterse et al., 2009). Besides NFs, rhizobia use the type 3 secretion system (T3SS) to suppress MTI by secreting type III effectors; the same system is employed by pathogenic bacteria for functioning in terms of virulence (Zamioudis and Pieterse, 2012). The interaction between flavonoid and NodD was found to activate *ttsI* expression, a type III secretion gene transcriptional activator, and this results in production of effector molecules [nociception receptor (Nop) proteins] that suppress host immunity (Okazaki et al., 2013; Gourion et al., 2015). A schematic representation of rhizobial mutualism and plant immunity modulation is presented in Figure 5.

MAMP-triggered immunity elicited against non-symbiotic beneficial microbes was found to be suppressed during the early stage of colonization with the help of MAMPs such as LPS and effector molecules such as EPS (Zamioudis and Pieterse, 2012). Given that ET signaling is an important factor in MTI, some bacteria target ET-dependent defense mechanisms by secreting the 1-aminocyclopropane-1-carboxylate (ACC) deaminase enzyme that targets ACC, the precursor of ET, to suppress ET production in plants (Glick et al., 2007; Millet et al., 2010). The other mechanism employed by non-mutualistic microorganisms is the elevated production of phytohormones; this mechanism has been reported to negatively cross talk with the prime SA-signaling pathway and affect the immune response (Verhage et al., 2010).

The induced JA/ET signaling pathway plays an important role in ISR induced by beneficial microorganisms, whereas the SA-induced pathway plays a similar major role in SAR induced by pathogens. However, some questions, such as how the plant permits rhizobial growth to generate an immune defense response, still need to be resolved. Rhizobial colonization shares few similarities with pathogen infection. Rhizobia use the T3SS to modify their host range, and the same process is identified with pathogenic bacteria in terms of virulence function. As observed in the case of the mycorrhizal association, the induced resistance in rhizobial association may be the after effect of induced MTI, wherein rhizobial microbes utilize some negative responses against elevated SA and ET production to establish the association. Simultaneously, they do not exert any adverse effect on the JA-dependent signaling pathway, indicating that JA plays a major role in ISR as well as in imparting systemic immunity in distal cells (Zamioudis and Pieterse, 2012; Singh et al., 2016). Several rhizobacteria have been reported to induce ISR against insect herbivores in plants (Zebelo et al., 2016).

## Perspective

During the course of their lifetime, plants are exposed to pathogenic microbes such as fungi, oomycetes, bacteria, phytoplasmas, nematodes, and viruses. Regardless of these continuous hostilities with pathogenic microbes, effective colonization is the exception rather than the rule in the natural world owing to the strength of plant immune systems (Piasecka et al., 2015). Plants are also continuously interacting with beneficial microbes, which also make an important contribution to plant defense activity. Plants are the ultimate energy source for most organisms; therefore, they have evolved highly efficient survival strategies with a strong immune system that is activated constitutively or after pathogen induction (Panstruga et al., 2009).

Although chemical control agents like fungicides and pesticides can efficiently fight against plant pathogens, they constitute severe environmental hazards. Therefore, the ecofriendly biocontrol strategies are becoming widely accepted promising alternatives to promote plants to fight pathogens. The plant-associated microbe dependent biocontrol strategies efficiently suppress pathogen by either directly antagonizing pathogens or enhancing plant resistance. Encouraging the colonization of antagonistic microorganisms in the premises of the plant can inhibit pathogen attack by producing antimicrobial compounds or by niche exclusion.

Application of beneficial microbes can induce rapid and strong immune responses in plants without triggering responses against themselves. These immune responses include induction of defense related metabolites in plants, which suppress plant pathogen. However, detailed studies are warranted to understand the mechanism underlying beneficial microbe-induced plant immunity and pathogen-induced immunity for the development of suitable biocontrol agents.

Exploitation of natural plant defense mechanisms provides novel methods for achieving better disease management. Enhanced production of PRRs and integration of R proteins in plants via engineering can boost microbe recognition ability of plants. Multiple disease resistant plant varieties can be generated by joining the integrated proteins from various NLRs, which recognize different effectors, into a single NLR. Overexpression of polygalacturonase inhibitor proteins (PGIT) or xylanase inhibitor proteins (XIP) in plants can induce resistance by acting against the pathogen producing virulence factors such as polygalacturonase or xylanase, respectively (Sun et al., 2018; Zhu et al., 2019). Overexpression of intermediate components of defense signaling pathways is another method for enhancing plant immunity. Overexpressions of hydrolytic enzymes in plants, which target microbial cell wall, and secondary metabolites, which act as antimicrobial components, are other widely accepted strategies.

Application of plant-based antimicrobial secondary metabolites is another promising strategy for pathogen control. Metabolite production and extraction is more convenient in suspension culture than the whole living plant. Hence large-scale production of secondary metabolites in plant suspension culture by manipulating biochemical pathways via metabolic engineering and their application on plants in the field can overcome negative impact of chemical control.

## Author Contributions

RN and TA conceived and wrote the manuscript. VR and AK contributed in editing and revising the manuscript. All authors contributed to the article and approved the submitted version.

## Conflict of Interest

The authors declare that the research was conducted in the absence of any commercial or financial relationships that could be construed as a potential conflict of interest.
